# The National Immunization Technical Advisory Group in Israel

**DOI:** 10.1186/s13584-021-00442-4

**Published:** 2021-01-26

**Authors:** Chen Stein-Zamir, Shmuel Rishpon

**Affiliations:** 1 The Israeli NITAG, Jerusalem, Israel; 2 Jerusalem District Health Office, Ministry of Health, Jerusalem, Israel; 3 The Hebrew University of Jerusalem, Faculty of Medicine, Braun School of Public and Community Medicine, Jerusalem, Israel; 4 Haifa District Health Office, Ministry of Health, Haifa, Israel; 5 School of Public Health, Faculty of Welfare and Health Sciences, University of Haifa, Haifa, Israel

**Keywords:** National Immunization Technical Advisory Group (NITAG), Routine immunization schedule, Decision-making, Evidence-based policy, Health policy

## Abstract

National Immunization Technical Advisory Groups (NITAGs) are defined by the World Health Organization as multidisciplinary groups of health experts who are involved in the development of a national immunization policy. The NITAG has the responsibility to provide independent, evidence-informed advice to the policy makers and national programme managers, on policy issues and questions related to immunization and vaccines.

This paper aims to describe the NITAG in Israel. The Israeli NITAG was established by the Ministry of Health in1974. The NITAG’s full formal name is “the Advisory Committee on Infectious Diseases and Immunizations in Israel”. The NITAG is charged with prioritizing choices while granting maximal significance to the national public health considerations. Since 2007, the full minutes of the NITAG’s meetings have been publicly available on the committee’s website (at the Ministry of Health website, in Hebrew).

According to the National Health Insurance Law, all residents of Israel are entitled to receive universal health coverage. The health services basket includes routine childhood immunizations, as well as several adult and post - exposure vaccinations. The main challenge currently facing the NITAG is establishing a process for introducing new vaccines and updating the vaccination schedule through the annual update of the national health basket. In the context of the annual update, vaccines have to “compete” with multiple medications and technologies which are presented to the basket committee for inclusion in the national health basket. Over the years, the Israeli NITAG’s recommendations have proved essential for vaccine introduction and scheduling and for communicable diseases control on a national level. The NITAG has established structured and transparent working processes and a decision framework according to WHO standards, which is evidence-based and country-specific to Israel.

The recent global COVID-19 pandemic is a major concern for all countries as well as a challenge for NITAGs. Currently, the NITAGs have a key role in advising both on sustainment of the routine immunization programs and on planning of the COVID-19 vaccination campaigns, with ongoing updates and collaboration with the Ministry of Health and health organizations.

## Introduction

According to the World Health Organization (WHO) guiding prerequisites, the National Immunization Technical Advisory Groups (NITAGs) are defined as multidisciplinary groups of national experts who are involved in the development of a national immunization policy. The NITAG has the responsibility to provide independent, evidence-informed advice to the policy makers and national programme managers, on policy issues and questions related to vaccines and immunization [1]. The Global Vaccine Action Plan (GVAP) was endorsed by the 194 Member States at the World Health Assembly in May 2012, as a framework to prevent millions of deaths by the year 2020, through improvement of equitable access to existing vaccines for people in all communities. The GVAP also called for all countries to establish or have an access to a NITAG by the year 2020 [2]. The GVAP criteria for NITAGs functionality include the following: legislative or administrative basis for the advisory group, formal written terms of reference, at least five different areas of expertise represented among core members, at least one meeting per year, circulation of the agenda and background documents at least 1 week prior to meetings and mandatory disclosure of any conflict of interest. In 2017, 98 countries reported on a NITAG which meets the GVAP functionality criteria, to provide guidance and counselling to the national immunization policies. According to the WHO-UNICEF Joint Reporting Form in 2019, 170 countries reported on a NITAG with 123 meeting the GVAP functionality criteria [3].

On a global level, the recommendations issued by WHO are formatted by the Strategic Advisory Group of Experts (SAGE) on Immunization established by the WHO Director-General in 1999. The SAGE is the principal advisory group to WHO for vaccines and immunization. It is charged with advising the WHO on overall global policies and strategies, ranging from vaccines and technology, research and development, to delivery of immunization and its linkages with other public health programs and preventive interventions. The SAGE is concerned not merely with childhood vaccines and immunization, but all vaccine-preventable diseases and all population groups [4]. These wide-spectrum responsibilities practiced by the WHO SAGE on immunization naturally influence the roles of local NITAGs. Globally, NITAGs aim to provide a basis for national governments to use in evidence-based decision making on vaccine and immunization policy. Yet, considerable variations have been reported between NITAGs including the legal basis, size and scope of committee membership, scope of work, the Ministry of Health role on the NITAG, existence of conflict of interest policies, and ultimate role in the decision-making process [5–7]. The goal of this integrative article is to portray the structure, status, routine working procedures and the challenges of the National Immunization Technical Advisory Group in Israel.

### The National Immunization Technical Advisory Group (NITAG) in Israel

#### Terms of reference

The Israeli NITAG was established by the Ministry of Health in 1974. The NITAG’s full formal name is “the Advisory Committee on Infectious Diseases and Immunizations in Israel”. According to the terms of reference, the Advisory Committee on Infectious Diseases and Immunizations advises and provides recommendations to the Director of Public Health Services, the Ministry of Health epidemiology division and the Ministry of Health heads, on communicable diseases control issues, with emphasis on vaccine preventable diseases (VPDs). All the NITAG processes are carried out with particular attention to safety issues.

The core goals of the NITAG recommendations are to decrease infectious diseases incidence and specifically the incidence and disease burden related to Vaccine Preventable Diseases, and to advocate for measures and public health programs aiming to increase and sustain safety and equity of vaccines utilization. Mainly, NITAG recommendations include the following issues:

1. Surveillance of infectious diseases and monitoring of incidence trends.
2. Utilization of screening methodology and screening tests for infectious diseases.
3. Control of sporadic, endemic and national infectious diseases outbreaks.
4. Recommendations on routine immunization schedules for children and adults.
5. Recommendations on special immunizations in specific circumstances.
6. General recommendations on vaccine use (e.g. vaccination age, number of vaccine doses, intervals between vaccine doses, precautions and contra-indications) as well as recommendations on specific vaccines.
7. Recommendations on utilization of unregistered vaccines or use of registered vaccines in circumstances different from the formal recommendation, if necessary.
8. Cost-effectiveness analyses on use of vaccines and vaccination programs.

The NITAG is expected to prioritize choices while granting high significance to the national public health considerations. In certain circumstances, such prioritization may lead to NITAG’s recommendations that are not entirely identical to those provided by the vaccine manufacturers. Attributable to the local epidemiological circumstances and considerations, the NITAG has previously recommended on specific scheduling and the number of vaccine doses. These recommendations were supported by national and international data and knowledge accumulated from the relevant scientific literature on these vaccines. Thus, in the introduction of the 7-valent pneumococcal conjugated vaccine (PCV7) into the national immunization program in 2009, the NITAG recommended a 2 vaccine doses + 1 booster vaccine schedule (at ages 2, 4 and 12 months) for PCV7 in infants, while the schedule recommended by the vaccine manufacturer was a 3 vaccine doses + 1 booster (at ages 2, 4, 6 and 12 months). Additionally, the NITAG recommended a 2-dose PCV7 supplementary program for toddlers in the second year of life. The introduction of the PCV7 to the national immunization program (2009) was followed by a marked decline in the incidence of invasive pneumococcal disease caused by PCV7 serotypes. In 2010, the PCV13 (13-valent pneumococcal conjugated vaccine) replaced the PCV7 in the national program. Currently, many countries also use the 2 + 1 doses vaccination schedule for the PCV13 [8, 9]. Another instance was at the introduction of the HPV (Human Papilloma Virus) vaccine into the national routine vaccination schedule. In 2013, HPV vaccine was included in the school-based vaccination program for 8th grade girls (age 13–14 years) in a 3-dose schedule (at 0, 1 or 2 months, and 6 months). In 2015, the HPV vaccination program was extended to include both 8th grade girls and boys in a 2-dose schedule (thus allowing for program expansion while reducing the additional costs). It is to be noted that currently the HPV vaccine manufacturer also recommends the 2-dose vaccination schedule [10].

#### NITAG committee members and participants

The Committee members come from various professions, affiliations and backgrounds. The committee includes public health physicians, epidemiologists, internal medicine and family medicine specialists, pediatricians, infectious diseases specialists, microbiologists and public health nurses. Legal counseling and economic expert guidance are available based on necessity. The NITAG members come from the Health Maintenance Organizations (or health funds), the hospitals, the universities and research institutions, the Ministry of Health and the Medical Corps of Israel Defense Forces. The broad spectrum of the NITAG members’ proficiencies enable the committee to address various issues (vaccine recommendations, schedules and prioritization) that require both scientific expertise on vaccines and expertise on public health policy matters (e.g. infrastructure, logistics and public attitudes). Currently, the committee consists of 15 core members, 9 ex-officio members and 3 observers. The NITAG participants are personally nominated by the Director of Public Health Services in the Ministry of Health. The processes of recruitment of potential members are informal and based on candidates’ background, professional knowhow and research qualities. The NITAG membership nomination is effective for a term of 5 years and can be extended. The Head of the NITAG committee for the last 25 years is a public health physician. Vaccine manufacturers are not represented in the committee; however, the manufacturers’ representatives may be invited to present data to the NITAG on specific issues and may not participate in the NITAG discussions. All NITAG members agree to participate on a voluntary basis and hence do not receive any payment, except for reimbursement of travelling expenses. The committee meetings are usually scheduled several times a year, while some meetings are conducted face-to-face, many meetings are organized as telephone or video conferences, mainly due to technical and logistic circumstances.

#### NITAG discussions and voting process

All NITAG members have the right to vote, including ex-officio members who represent the Ministry of Health. Recommendations are decided by consensus or by the members’ voting. The NITAG has no permanent working groups. Special ad-hoc working groups have been nominated over the years when considered necessary (e.g. working groups on herpes zoster vaccines, on conjugated meningococcal vaccines and on establishing an updated case definition for pertussis surveillance). The NITAG meets at the request of the committee’s head or members or regrading issues raised by the Ministry of Health epidemiology division, Public Health Services or the Ministry of Health Director General. The basic processes for the committee discussions and voting are:

1. Data on the disease and prevention are presented by the Ministry of Health epidemiology division and by appropriate invited experts on the subject matter.
2. International recommendations (mostly English-language) of CDC, ECDC, Health
  Canada, JCVI and WHO SAGE. The committee members review the recommendations and evaluate their applicability as to the Israeli data and the national health system infrastructure.
3. Cost-effectiveness, cost-benefit and cost-utility evaluations and considerations (either Israeli-based or retrieved from other countries) are included when available. However, in many discussions, the complete cost-effectiveness data and models specifically relevant to the Israel are unattainable.
4. Accepted public health practices in other developed countries (OECD).
5. Public health values and ethical principles especially those referring to equality, Equity and solidarity.

#### NITAG recommendations

Overall, the committee’s recommendations are highly respected by the Ministry of Health, the leaders of the health system in Israel, the health professionals and the health organizations. The Ministry of Health adopted almost all of the recommendations issued by the NITAG committee. However, some of the applications and specifically the inclusion of new vaccines into the national public health basket have been postponed, mainly due to budgetary restrictions [11, 12]. The main issues which have been on Israel’s NITAG agenda during the last decade (years 2010–2019) are presented in Tables 1 and 2, with the main NITAG recommendations. The accepted recommendations appear in Table 1 and those recommendations that, to date, had not been accepted appear in Table 2.

**Table 1:** Israel’s NITAG meetings for the years 2010–2019 – Recommendations accepted

Year	Main issues discussed by NITAG	Recommendations
**2010**	Rubella vaccination program evaluation	No change in the program
	Priorities in adding new vaccines to the routine vaccination program	1st priority: Influenza vaccination of young children < age 5 years. Accepted 2011. 2nd: Tdap for women after delivery
**2011**	Meningococcal Vaccines (international travel)	Preference for conjugated Meningococcal Vaccines
	Pneumococcal vaccination policy for infants	Continue the successful Pneumococcal vaccination program
	Influenza vaccination policy	Recommending universal Influenza vaccination policy (aimed at all population groups aged 6 months and above).
	Information on vaccines safety to the public	How to present information on vaccines safety to the public? Recommended: use international publications by WHO, CDC, ECDC etc.
	Herpes Zoster vaccine	Recommended for persons 65 years old and above 2012 - Added to the national health basket in 50% discount
	HPV vaccines: 4 valent vs. 2 valent	Preference for the 4 valent HPV vaccine.
	Rota Virus vaccine	Introduction of Rota Virus vaccine into the national routine immunization schedule, 2011
**2012**	Use of PCV10 or PCV13	Continue the PCV13 in the national childhood vaccination program
	Measles elimination strategies	Adopting the measles elimination committee recommendations for disease surveillance and for improving and sustaining the measles vaccination coverage
**2013**	Polio vaccination policy	During the “silent” WPV1 event in Israel (isolation in sewage in southern Israel, mainly Arab Bedouin localities). Supporting the vaccination campaigns Adding 2 bOPV vaccine doses at ages 6, 18 months to the schedule.
	Tdap vaccine for pregnant women	Tdap vaccine recommendation accepted for women in the third trimester of pregnancy. Applied in 2015.
	PCV 13 for use in adults	Recommended for adults in defined high-risk groups, Applied in 2016
	HPV vaccines – safety review	HPV vaccines safety review established before introduction into the routine immunization program in schools: 2013 – HPV vaccine for females 8th school grade
**2014**	Polio elimination	Adopting the polio elimination committee recommendations for continuation of the polio virus environmental sewage sampling program and preparing an outbreak preparedness plan. Keeping the bOPV vaccine at 6 and 8 months in the routine schedule.
**2015**	HPV vaccination policy	Recommendation for a 2-dose HPV vaccination schedule 2015- females and males in the 8th school grade
	Rotavirus vaccination provision in neonatal units	Continue previous recommendation not to vaccinate infants while hospitalized in neonatal intensive care units (prolonged stay).
	Priorities of introduction of new vaccines	Recommended: Meningococcal vaccines and Hepatitis A vaccines for high risk groups. Applied in 2016
	Influenza vaccination policy	Preference of LAIV use in children
**2016**	Vaccination of the elderly - PCV	PCV not indicated to healthy persons 65+ years.
	Vaccination of the elderly - Herpes Zoster vaccine	Herpes Zoster vaccine inclusion recommended to the basket of services committee. Included 2017 with price reduction of 50%
	Influenza vaccination policy	Vaccination of children in elementary schools. Program introduced in 2016–2017. Preference for 4 strains Influenza vaccines. (2019: 4 strains)
	Pregnancy after MMR	Ratification of the recommendation indicating no need for interval between MMR vaccination and pregnancy.
**2017**	Definition of immunity status against measles	Requiring written proof of measles vaccination status (except travelers). Validity of immunity based on ELISA antibodies tests
**2018**	MenB vaccination policy	Vaccination of high risk groups (included in the health basket 2020)
	Neonatal BCG vaccination policy in risk groups	Continue vaccination of neonates whose parents come from countries of high TB endemicity
	National measles outbreak - Mandatory vaccinations	Mandatory vaccinations and providing proof of vaccination before school entry were not recommended. In hyperendemic areas, unvaccinated children were banned from affected schools and kindergartens.
	National measles outbreak - vaccination in schools	Prioritization of measles vaccine over HPV and influenza vaccines. Applied 2018–2019
**2019**	Influenza vaccines	3 or 4 strains, adjuvanted vaccines. NITAG recommended 4 strains.
	Prioritizing Vaccinations to the national health basket	MMR vaccine for the adult population, Updating schools’ HPV vaccine program (9 valent replaced 4 valent), MenB vaccine for high risk groups. Included in the health basket 2020

**Table 2:** The NITAG meetings in the last decade in Israel 2010–2019 – recommendations not accepted

Year	Main issues discussed by NITAG	Recommendations
**2010**	Mumps outbreak 2009–2010	MMR vaccine dose catch-up to all schoolchildren (2nd to 9th grades)
**2012**	Pertussis vaccination of adults	Preference for Tdap vaccine over Td vaccine
	HPV vaccination policy	HPV catch-up vaccinations for 15–26 years old females
**2014**	HPV vaccination policy	Re-recommendation for HPV catch-up program
	Meningococcal serotype B vaccine	Men B vaccine in the national program. Relevant data needed, on the preventive system capacity to comply with the requirement for additional vaccination visits.
**2017**	Vaccinations for the national health basket	Herpes Zoster vaccine for 65 years old and above
**2018**	Measles vaccination policy	MMR 2nd dose at age 2 years instead of 1st grade of school
	Screening for HBV	HBV screening for persons before chemotherapy initiation
	Meningococcal serotype B vaccine	Including Meningococcal serotype B vaccine in the national routine vaccination program.
	Vaccinations for the national health basket	Herpes Zoster vaccine for 65 years old and above
**2019**	Measles vaccination policy	Re-recommendation for MMR 2nd dose at age 2 years.

#### Transparency of discussions

Since 2007 the full minutes of NITAG’s meetings, including the named citations of each speaker in the discussions are available on the NITAG website (Ministry of Health website, in Hebrew) [13]. The NITAG head had started this public transparency initiative based on recommendations of the WHO Expanded Program on Immunization (EPI) managers meeting in Dubrovnik 2007. To date, the high transparency level of the NITAG’s meetings protocols does not appear to cause concerns over the years.

#### Conflict of interests

All NITAG nominees receive a written document regarding conflict of interests, as part of the committee nomination documents. Nominating committee members is carried out while striving to achieve the highest level of professional expertise possible, as well as minimizing real or potential conflict of interests. Members who manage vaccine clinical trials or who participate in data and safety monitoring boards of clinical trials may advise the NITAG and present data on these vaccines, but they may neither participate in the discussions nor vote on subjects related to these particular vaccines. With regard to other vaccines developed or produced by the same manufacturer, those members may participate in the discussion, but do not vote on the decisions. As a rule, the NITAG head opens all meetings with a request to all members to declare potential conflict of interests on issues in the meeting agenda.

#### Other committees on immunizations and infectious diseases

Other committees on Immunizations and Infectious Diseases are the national Epidemic Management Team (EMT) for emergency outbreaks and epidemic threats (e.g. Influenza pandemic, bioterrorism, COVID-19 pandemic), specific committees nominated for disease elimination verification as required by WHO (e.g. polio, rubella, measles), the national advisory committees on HIV/AIDS and on Tuberculosis and on international travel recommendations. Currently, the Epidemic Management Team and the NITAG are engaged in a joint discussion on the recommendations and strategies regarding the introduction of the novel COVID-19 vaccines.

#### The NITAG reorganization

The committee considered the recommendations regarding NITAG’s operation published in 2010 [5–7] and the SIVAC initiative (Supporting Independent Immunization and Vaccine Advisory Committees) on ameliorating the structure and function of the Committee [14]. The Israel NITAG reorganization, which took place in 2012, included the following components. The committee’s term of office was limited to a period of 5 years (previously it had not been limited), with the possibility of adding more terms. The committee, which had been composed of only one group of members, now includes three groups: core members, ex-officio members and observers. The core and ex-officio NITAG members have voting rights. The NITAG observers are potential future core members. The services of a health economist and a legal counsellor became available to the committee. The committee adopted updated and detailed Terms of Reference and supplemented the guidelines on Conflict of Interests.

## Discussion

The WHO encourages all countries to promote establishment and strengthening of National Immunization Technical Advisory Groups (NITAGs) that provide recommendations on immunization policies and programs (e.g., vaccination schedules, improvements of routine immunization coverage, new vaccine introduction, etc.) [5–7, 14]. The NITAG main goal is provision of immunizations recommendations, which are evidence-based and country-specific.

The GRADE methodology (Grading of Recommendations’ Assessment, Development and Evaluation) working group has become the recommended operational mode based on quality of evidence [15, 16]. The GRADE methodology principals are considered operational in the NITAGs’ working process. Similar to other NITAGs in developed countries, the key factors considered in the decision-making process of adopting vaccines in the national immunization program in Israel include several components. These include disease burden, severity and consequences, vaccine safety and immunogenicity, vaccine-efficacy and effectiveness models, feasibility issues, priority among VPDs, logistics and method of vaccine administration, economic evaluations, international recommendations (WHO, CDC, ECDC) and public perceptions on diseases and vaccines [16]. While not all the desired information is available to the NITAG, genuine efforts are persistently made to provide the most updated data.

Over the years the NITAG in Israel has been actively involved in many broad-spectrum national public health policy discussions and recommendations, including, for example, the NITAG participation in setting objectives for “Healthy Israel 2020”. The committee provided significant support in the establishment process of the national immunization registry in Israel [17] and participated in the recent discussion concerning a public initiative to require the presentation of the child’s personal vaccination record upon the admission to kindergartens and schools [18].

All residents of Israel are entitled to receive universal health coverage since the introduction of the National Health Insurance Law (NHIL) in 1995 [19]. The NHIL basket of health services contain vaccines included in the routine childhood immunization schedule for infants, toddlers, and schoolchildren, several vaccines for adults and vaccines that are exposure-related (e.g. Rabies Vaccines). Vaccinations of Health Care Workers are part of the employing organizations responsibilities. International travel vaccinations require individual out of pocket co-payment.

A major challenge that Israel’s NITAG currently faces concerns introduction of new vaccines into the health basket. Prior to the NHIL the procedure of introducing vaccines into the schedule was based on the following: provision of NITAG recommendations, decision-making by the Public Health Services and the Ministry of Health and application to the Ministry of Finance for allocation of appropriate budgeting. The national childhood immunization schedule was updated regularly according to international guidelines. In certain vaccines, Israel has been leading, being the first nation globally to introduce a universal Hepatitis A vaccination program for toddlers in 1999 and provide real-world high effectiveness data as well as evidence of herd immunity [20]. Since the NHIL era the procedure of including new vaccines in the health basket has undergone several phases and alternations. In 1999–2007, new vaccines recommended by the NITAG had not been incorporated into the national health basket. These vaccines (recommended in line with global recommendations) included Pneumococcal Conjugate Vaccine, Rota virus vaccine, Varicella vaccine (at 12 months and the first school grade), Tdap vaccine in 8th grade and HPV (Human Papilloma Virus) vaccine for schoolgirls in 8th grade. In 2007, the Ministry of Finance approved budgeting for these vaccines in a 5-years plan [21]. The plan led to positive public health outcomes; introducing Varicella vaccine (2008), Pneumococcal Conjugate Vaccine (2009) and Rota virus vaccine (2011) into the immunization program was followed by reduction in incidence and disease burden [8, 22, 23]. The Rota virus vaccine was the first vaccine to enter the national health basket, after a process of NITAG recommendation and approval of the national Public Committee for the Expansion of the Medical Health Basket Services [23, 24].

The updating of Israel’s national health basket in is a comprehensive, systematic and long-standing procedure. Each year hundreds of new medical technologies applications are presented to the national Public Committee for the Expansion of the Medical Health Basket Services that has to decide which technologies to include given a defined budget [25–27].. While the decision making process is both evidence-based and transparent, efficiency preferences seem to be given priority to equity concerns [27]. Addition of preventive public health measures such as vaccines into the basket is hence perceived as less urgent than life saving medical technologies aimed at specific patients and disease conditions. In the annual update process (since 2011) vaccines have to “compete” with multiple medications and technologies. The conflict between inclusion of essential lifesaving and urgent medications and technologies and of preventive public health measures such as vaccines is inevitable. Several applications issued by the NITAG to the MoH to promote a defined “vaccination basket” annual national budget allocation were unsuccessful.

Another challenge is the vaccine-specific receipt rates after the NITAG’s recommendations acceptance. The routine vaccination coverage rates in Israel’s schools for the diphtheria, tetanus, and acellular pertussis (Tdap) and the measles, mumps, rubella, and varicella (MMRV) vaccines are traditionally high and both above 95%. However, the reported coverage rates for the HPV and the influenza vaccines in schools are lower [28]. HPV vaccine for 8th grade schoolgirls and schoolboys entered the health basket in 2013 and 2015, respectively [29]. The HPV vaccine coverage differ among population groups in Israel. The HPV vaccination coverage rates in schools are about 60% overall, yet, the rates in Arab schools (over 80%) and secular Jewish schools (70–80%) are higher compared to orthodox Jewish schools (range 0–35%) [28, 30, 31]. The schools’ Influenza vaccination program had been recommended by the NITAG for students in the 1st-6th grades. As to budgetary and logistic limitations, the program had been implemented gradually. Influenza vaccination was introduced in the 2nd grade (2016–17) and in 3rd and 4th grades (2017–2018). In the 2018–2019 school year, the overall influenza vaccine coverage rates were 45, 36 and 30% for the 2nd, 3rd and 4th grades, respectively [28]. Health promotion programs seem necessary to improve the vaccination coverage for these vaccines. School-based vaccinations programs are operated in many countries with the most common vaccines being tetanus, diphtheria and HPV vaccines [32]. A school-based influenza vaccinations programme was first implemented in the United Kingdom in 2013, in a gradual process, with increasing coverage rates (80.5% in 2018–2019) and a positive impact on influenza-related outcomes [33].

Adult vaccines introduced in recent years include the pertussis vaccine in the third trimester of pregnancy (2015) with an estimated 75% coverage [34]. Pertussis patterns modeling (1998–2019) before and after 2015, showed decline in pertussis incidence (71%) and hospitalizations (58%) among infants aged 2 months and younger, probably associated with maternal vaccination [35]. The pneumococcal conjugated vaccine (PCV) for high-risk groups was included in the health basket in 2016, by the MoH, based on the US Advisory Committee on Immunization Practices (ACIP) recommendations. The high-risk groups include individuals with impaired splenic function, immunocompromising conditions (including HIV), cochlear implant placement or cerebrospinal fluid leak. The NITAG has discussed the vaccination policy for persons aged 65 years and above and decided to keep the recommendation for pneumococcal polysaccharide vaccine (PPV23) and not recommend PCV in this group. This recommendation was based on data on the decline in pneumococcal disease incidence (vaccine serotypes) observed in Israel, since the introduction of PCV into the infants’ routine vaccination schedule in 2009 [8].

Another challenge faced by the NITAG while considering adding new vaccines concerns public health infra-structure issues and logistic feasibility. It is indeed controversial whether the NITAG vaccine introduction recommendations should be “purely” professional or otherwise take into account also practicability and logistic aspects. The preventive health services offered to Israel’s children in Maternal Child Health Clinics (MCHC) are highly regarded and provide universal programs with routine vaccinations provided to all children free of charge. However, these services suffer from staff shortages and insufficient budgeting to cope with the constantly increasing number of children and the new tasks [36, 37]. Introducing the Meningococcal B Vaccine (MenB) vaccine into the routine schedule required adding several MCHC visits, so that it can be provided separately from other vaccines, to reduce the probability of fever. Thus, MenB vaccine has not been included in the routine immunization schedule provided free of charge at the community MCHCs (Table 2, 2018). The MenB vaccine for children in Israel is currently available with parental co-payment only through the health funds complimentary insurance [38].

Similarly, mainly attributable to budgetary constraints, during the years 2016–2018 applications of NITAG recently recommended vaccines for adults, the herpes zoster vaccine for the elderly population and the Tdap vaccine for adults, were repeatedly unsuccessful regarding the inclusion of these vaccines in the national health basket (Table 2).

In the last decade, the NITAG has been engaged in outbreak containment activities and programs nationally. Israel reported to the WHO in June 2013 on wild poliovirus type 1 (WPV1) isolation in environmental sampling of sewage in southern Israel (mainly Arab Bedouin localities). The WPV1 shedding was found mainly among young children, without any clinical paralytic polio cases. The effective polio control measures included augmented disease monitoring (clinical and environmental) and mass vaccination (IPV catch-up and bOPV, bivalent OPV, Sabin 1 and Sabin 3 polio vaccine strains, the “2 Drops” campaign) [39]. The NITAG recommended that following the introduction of bOPV vaccine in 2013 it will be included in the national routine childhood immunization schedule, now consisting of IPV and bOPV (Table 1). Despite a long standing 2-dose measles vaccination plan with high overall vaccination coverage, a large measles outbreak (4300 notified cases) emerged in Israel in 2018–2019 following virus importations and controlled by measles vaccination campaigns [40–42]. During the measles outbreak the NITAG discussed proposals for mandatory vaccinations for children and/or providing proof of vaccination before school entry, and had not recommended use of mandates. In regions with high measles endemicity, unvaccinated children were banned by the district health officers from affected schools and kindergartens during local measles outbreaks. The NITAG recommended that Maternal Child Health Clinics and school health services will prioritize the MMR vaccines over other activities (Table 1). The NITAG proposed enhanced surveillance, a communications plan for health care workers and the general public and focusing on under-vaccinated groups (e.g. Jewish orthodox) [43, 44]. Regarding vaccination policies, the NITAG, in collaboration with the measles elimination committee, adopted the WHO measles elimination framework for obtaining and sustaining high vaccination coverage rates nationally (the “catch-up”, “keep-up”, “follow-up” and “mop-up” vaccination campaigns) [43, 45]. Hence, the NITAG has graded its 2020 health basket recommendations, giving the highest priority to adults’ measles vaccinations (Table 1). The NITAG recommended a catch-up program of a 2-dose measles vaccination for adults aged 18 years and above and born after 1957 to be included in the national health basket and provided by the health funds. Despite approval, the program had not started due to the 2020 COVID-19 pandemic. Regarding routine measles vaccination schedule (first dose at 12 months and second dose at 6 years) the NITAG suggested providing the second measles vaccine dose at age 2 years; this proposal has not been applied [43].

Major challenges of another category facing the public health system and involving the NITAG are vaccination acceptance and vaccination coverage rates. While the overall childhood vaccination coverage in Israel is conventionally high, under-vaccination and vaccination delays have been documented among specific population groups [44]. The World Health Organization has recently defined vaccine hesitancy as one of the ten major threats to global health in 2019 and as an emerging crisis ranging from hyperlocal to national and global scale. Vaccine-Preventable Diseases outbreaks often originate in communities with sub-optimal immunization coverage rates but may spread rapidly across borders. The NITAGs should be involved in formatting public health action plans which are evidence-based and multi-disciplinary [46, 47]. Israel’s NITAG has discussed vaccine hesitancy several times and recommended on programs to improve the accessibility and availability of preventive services to all children and on application of Tailored Immunizations Programs (TIP) based on a culture-sensitive approach. The NITAG has debated a mandatory vaccinations policy without recommending it. Notably, the definition of mandatory vaccinations is not globally standardized. A recent study in 28 NITAG countries globally showed marked diversity, about half the countries indicated mandatory elements in the national program with variability in mandated vaccines and use of sanctions [48].

Formation of national immunization policy by governments is supported by the independent, structured and evidence-informed recommendations and guidance provided by the NITAGs [49]. The issue of allocated budgeting and the part of the Ministry of Finance have been described as highly influential regarding if, when and how NITAG recommendations are adopted and implemented [50]. The NITAGs worldwide vary as to their affiliation and proximity to the national government, mainly to the Ministry of Health [5–7]. Israel’s NITAG discussions are mainly conducted on queries raised by the public health services of the Ministry of Health with certain affiliation to the government. The main advantage of distance from the government is ensuring the NITAG’s complete independence, which is a major criterion for issuing professional recommendations unaffected by the Ministry of Health constrains. The advantages of proximity to the government is reduction of possible disagreements, increasing trust and enabling shared perceptions between the NITAG and the Ministry of Health. Proximity to the government may also support and advance the probability of future acceptance of the NITAG recommendations and appropriate budget allocation.

The COVID-19 pandemic is currently a major global concern as well as a challenge for all NITAGs [51]. The NITAGs have a key role in advising both on sustainment of the routine immunization programs and on planning of the COVID-19 vaccination campaigns. These processes are carried out with regard to national and global references and recommendations, in close collaboration with the Ministry of Health policymakers and the leadership of health organizations. The crucial mission of making COVID-19 vaccine campaigns successful relies on the ongoing collaboration of health organizations and on proper, updated and transparent information presented to the public. The NITAG’s role in the exceptional COVID-19 pandemic challenges is essential.

## Conclusions

The Israeli NITAG’s recommendation have proved essential for vaccine introduction and scheduling and for communicable diseases control on a national level. The NITAG has established long-standing, structured and transparent working processes and decision framework according to the WHO standards. Future challenges include improving the introduction of new vaccines and updating the vaccination schedule through the national health services basket, strengthening the public health infra-structure and sustaining vaccination coverage.

## Data Availability

Data sharing not applicable to this article as no datasets were generated or analyzed during the current study.

## Abbreviations

BCG: Bacillus Calmette–Guérin vaccine
CDC: Centers for Disease Control and Prevention
ECDC: European Centre for Disease Prevention and Control
EMT: Epidemic Management Team
EPI: Expanded Program on Immunization
GRADE: Grading of Recommendations’ Assessment, Development and Evaluation
GVAP: Global Vaccine Action Plan
HPV: Human Papilloma Virus vaccine
JCVI: Joint Committee on Vaccination and Immunisation (UK)
MenB: Meningococcal serotype B vaccine
MMR: Measles-Mumps-Rubella vaccine
NHIL: National Health Insurance Law
NITAG: National Immunization Technical Advisory Group
PCV: Pneumococcal conjugate vaccine
SAGE: Strategic Advisory Group of Experts
SIVAC: Supporting Independent Immunization and Vaccine Advisory Committees
Tdap: Combined Diphtheria, Tetanus toxoid, and acellular Pertussis vaccine
TIP: Tailored Immunizations Programs
UNICEF: United Nations Children’s Fund
VPD: Vaccine-Preventable Disease
WHO: World Health Organization
